# Genomic and immunocyte characterisation of bloodstream infection caused by *Klebsiella pneumoniae*

**DOI:** 10.1186/s12941-024-00721-3

**Published:** 2024-06-20

**Authors:** Wei Yu, Chen Huang, Xiang Lian, Lushun Jinag, Hong Li, Ping Shen, Yonghong Xiao

**Affiliations:** 1 State Key Laboratory for Diagnosis and Treatment of Infectious Diseases, National Clinical Research Center for Infectious Diseases, Collaborative Innovation Center for Diagnosis and Treatment of Infectious Diseases, The First Affiliated Hospital, Zhejiang University School of Medicine, Hangzhou, China; 2 Department of Respiratory Medicine, Ningbo Medical Center Lihuili Hospital, Ningbo, China; 3 Department of Infectious Diseases, The Affiliated Xiangshan Hospital of Wenzhou Medical University, Xiangshan First People’s Hospital Medical and Health Group, Ningbo Fourth Hospital, Ningbo, China

**Keywords:** *Klebsiella pneumoniae*, Cytotoxic T lymphocytes, B lymphocytes, Virulence genes

## Abstract

**Objectives:**

The aim of this study was to evaluate the characteristics of immunocyte associated with bloodstream infection (BSI) caused by *Klebsiella pneumoniae* (Kpn).

**Methods:**

Patients with BSI-Kpn were included from 2015 to 2022 in our hospital. Immunocyte subpopulations of enrolled BSI-Kpn patients were tested on the same day of blood culture using multicolor flow cytometry analysis. Antibiotic susceptibility test was determined by agar dilution or broth dilution method. All included isolates were subjected to whole genome sequencing and comparative genomics analysis. Clinical and genetic data were integrated to investigate the risk factors associated with clinical outcome.

**Results:**

There were 173 patients with non-duplicate BSI-Kpn, including 81 carbapenem-resistant Kpn (CRKP), 30 extended-spectrum β-lactamases producing Kpn (ESBL-Kpn), 62 none CRKP or ESBL-Kpn (S-Kpn). Among 68 ST11-CRKP isolates, ST11-O2v1:KL64 was the most common serotypes cluster (77.9%, 53/68), followed by ST11-OL101: KL47 (13.2%, 9/68). Compared with CSKP group, subpopulations of immunocyte in patients with CRKP were significantly lower (P < 0.01). In patients with ST11-O2v1:KL64 BSI-Kpn, the level of cytotoxic T lymphocytes (CD3 + CD8 +) is the highest, while the B lymphocytes (CD3-CD19 +) was the least. In addition, the level of immunocyte in patients with Kpn co-harbored *clpV*-*ybtQ-qacE* were lower than that in patients with Kpn harbored one of *clpV*, *ybtQ* or *qacE* and without these three genes. Furthermore, co-existence of *clpV*-*ybtQ*-*qacE* was independently associated with a higher risk for 30-day mortality.

**Conclusions:**

The results demonstrate that patients with BSI-CRKP, especially for ST11-O2v1:KL64, exhibit lower leukomonocyte counts. In addition, BSI-Kpn co-harbored *clpV*-*ybtQ*-*qacE* is correlated to higher 30-day mortality.

**Supplementary Information:**

The online version contains supplementary material available at 10.1186/s12941-024-00721-3.

## Introduction

*Klebsiella pneumoniae* (Kpn) is one of significant pathogens for community-acquired and hospital-acquired infections, especially for bloodstream infection (BSI). In the last decade, the rate of BSI caused by carbapenem-resistant *K. pneumoniae* (CRKP) is trending upwards from 7.0% in 2014 to 19.6% in 2019 [1]. In addition, BSI-CRKP were associated with high mortality due to few therapeutic options [2, 3]. Recently, several novel antibiotics are available to combat CRKP, however, there have been reports of resistance to novel ß-lactams and ß -lactamase inhibitors [4, 5]. Therefore, clinical treatment for BSI-Kpn could not just rely upon antibiotics, and the interaction between CRKP and the host immune response needs to be further investigated.

Recent studies demonstrated that Kpn could enhance resistance by mediating cytokine levels and immune cell recruitment in host, resulting in disease-tolerant immune response [6–8]. In addition, several other evidence demonstrated that Kpn*,* especially for ST258 CRKP could actively subvert host defence by different immune evasion strategies, such as limiting C3b deposition, triggering apoptosis in macrophages and secretion of several siderophores [6]. Notably, ablating host defence by Kpn could cause a more pronounced bacteremia and accelerated mortality in infected mice [9, 10]. However, the majority of evidence on the interplay between Kpn and host immune response were obtained by infecting rodents, chiefly by mice [8–10]. Few studies addressed the response of immune cells to counteracting BSI- Kpn, especially for ST11 CRKP. Therefore, this present study was conducted to elucidate the immune cells response against different BSI-Kpn in clinic. In addition, clinical and genetic data were integrated to further investigate the risk factors associated with clinical outcome.

## Methods

### Patients and clinical data

A retrospective study was conducted to obtain profiles of the immune response in patients with BSI-Kpn and identify the risk factors associated with 30-day mortality. Clinical diagnosis of BSI-Kpn was screened by positive blood culture at The First Affiliated Hospital, Zhejiang University School of Medicine from 2015 to 2022. Meanwhile, patients tested immunocyte subpopulations on the same day of blood culture. The medical records of included patients were reviewed. The detailed analysis data and definitions were obtained as described in our previous study [11, 12]. The 30-day mortality was used as treatment outcomes. This study was approved by the recommendations of the Ethics Committee of The First Affiliated Hospital, Zhejiang University School of Medicine.

### Analysis of immunocyte subpopulations with flow cytometry

The immunocyte subpopulations in the whole blood, including mature peripheral T lymphocytes (CD3 +), helper T lymphocytes (CD3 + CD4 +), cytotoxic T lymphocytes (CD3 + CD8 +), B lymphocytes (CD3-CD19 +), and natural killer cells (CD3-CD16 + CD56 +), were tested by multicolor flow cytometry as a previous study [13]. Heat map analysis for cytokine data was performed using TBtools [14].

### Strain collection and antibiotic susceptibility test

A total of 173 BSI-Kpn strains were collected in the study. The minimal inhibitory concentration (MIC) of 20 antibiotics against BSI-Kp isolates were determined by agar dilution method, while antimicrobial sensitivity of tigecycline and polymyxin B was done by broth dilution method. Extended-spectrum-β-lactamase (ESBL)-producing isolates were confirmed for ESBLs production by disk synergy test [15]. CRKP was defined as Kpn strain resistant to imipenem or meropenem or ertapenem. Non-CRKP or non-ESBL-Kpn was considered as “susceptible” Kpn (S-Kpn). ESBL-Kpn and S-Kpn belong to carbapenem-sensitive *K. pneumoniae* (CSKP). The details of antibiotics were consistent with our previous study [16]. The results for test antibiotics were interpreted according to Clinical and Laboratory Standards Institute (CLSI) guidelines [15].

### Genome sequencing and comparative genomics analysis

The genomic DNA of isolates was extracted by QIAamp DNA Mini Kit (Qiagen, Hilden, Germany). Whole-genome sequencing (WGS) was performed on HiSeq 2000 platform (Illumina, USA) by the Beijing Novogene Institute (Beijing, China). Illumina raw reads were assembled and annotated using SPAdes v3.14.1 and Prokka v 1.10, respectively [17, 18].

Antimicrobial resistance genes, and virulence genes were identified using Resfinder and VFDB databases, respectively. Phylogenetic trees were built from core genome single nucleotide polymorphisms using kSNP4.1 [19]. K/O-type was determined using Kaptive v1.0 [20].

All sequence data has been deposited under BioProject accession No. PRJNA778807 and PRJNA1056658.

### Statistical analysis

The results for abnormal distribution of continuous variables were presented as median (interquartile range) and analyzed using chi-square test. Multivariate analysis was performed after identifying variables with a P-value of < 0.05 in the univariate analysis. A two-tailed P value < 0.05 was considered to be statistically significant. GraphPad Prism software (V8.0) and SPSS 23.0 for Windows (SPSS Inc., Chicago, IL, USA) were used to analyze data. The 30-day mortality was established basing on the regression model by employing the R v4.1.2 package.

## Results

### Clinical characteristics in patients with BSI-Kpn

There were 173 patients with non-duplicate BSI-Kpn, including 81 CRKP, 30 ESBL, 62 S-Kpn, were included in this study. The clinical characteristics of all patients are shown in Table 1. There were no significant differences in gender and age. Organ transplant patients were more likely to be infected with CPKP. In addition, CRKP patients also underwent more invasive procedures. The levels of albumin and procalcitonin in patients with CRKP were lower than that in patients with CSKP. More patients in CRKP group received aminoglycoside, fosfomycin, tigecycline, polymyxin B, and ceftazidime-avibactam. APACHE-II score and SOFA score in CRKP were higher than that in CSKP (P < 0.05). Furthermore, the 30-day mortality in CRKP was significantly higher than that in CSKP (P < 0.000).

**Table 1:** Clinical characteristics of all patients with BSI-Kpn

Parameter/category	CSKP (N = 92)	CRKP (N = 81)	P-value
Gender, male, N (%)	59 (64.1)	54 (66.7)	0.727
Age (years)	52.1 ± 15.7	52.7 ± 14.5	0.81
APACHE-II score	10.8 ± 5.7	12.7 ± 5.7	0.026
SOFA score	5.2 ± 4	7.4 ± 4.1	0.001
Prior chemotherapy or radiotherapy, N (%)	17 (18.5)	16 (19.8)	0.831
Prior corticosteroid therapy, N (%)	28 (30.4)	38 (46.9)	0.026
Prior immunosuppressant use, N (%)	19 (20.7)	30 (37)	0.017
Comorbid illness, N (%)			
Diabetes	23 (25.0)	10 (12.3)	0.035
Hepatitis	24 (26.1)	30 (37.0)	0.121
Tumor	23 (25.0)	22 (27.2)	0.747
Hypertension	34 (37.0)	26 (32.1)	0.503
Coronary heart disease	1 (1.1)	4 (4.9)	0.131
Cerebral infarction	4 (4.3)	4 (4.9)	0.854
Renal insufficiency	21 (22.8)	17 (21.0)	0.771
Trauma	4 (4.3)	7 (8.6)	0.248
Organ transplant	20 (21.7)	30 (37.0)	0.027
Invasive procedures, N (%)			
Mechanical ventilation	34 (37.0)	56 (69.1)	< 0.000
Hemodialysis	17 (18.5)	27 (33.3)	0.025
Urinary catheterization	46 (50)	62 (76.5)	< 0.000
Arterial catheterization	44 (47.8)	70 (86.4)	< 0.000
Stomach tube	34 (37.0)	55 (67.9)	< 0.000
Bronchofibroscope use	11 (12.0)	13 (16.0)	0.437
Wound drainage tube use	65 (70.7)	71 (87.7)	0.004
Laboratory examination			
White blood cell (10E9/L)	9.8 ± 7.1	8.3 ± 6.3	0.139
Neutrophil percentage (%)	82.7 ± 21.7	83.5 ± 21.4	0.805
Hemoglobin (g/L)	96.5 (78.3–125.5)	77.0 (65.0–90.5)	< 0.000
Platelet (10E9/L)	128.1 ± 111.2	100.0 ± 91	0.073
Hypersensitivity C reactive protein (mg/L)	95.1 ± 79.6	85.5 ± 66.5	0.39
Procalcitonin (ng/ml)	3.4 (0.8–16.1)	2.6 (0.8–9.5)	0.039
Albumin (g/L)	33.2 ± 5.8	34.8 ± 5.6	0.061
Alanine transaminase (U/L)	37.0 (16.3–96.5)	48.0 (17.0–169.5)	0.151
Aspartate aminotransferase (U/L)	40.0 (20.0–76.8)	45.0 (18.5–122.0)	0.482
Cholinesterase (U/L)	3941.8 ± 1894.2	3422.9 ± 1610.9	0.056
Total bilirubin (μmol/L)	99.1 ± 463.7	74.0 ± 99.8	0.633
Serum creatinine (μmol/L)	152.6 ± 213.9	152.0 ± 204.4	0.985
International normalised ratio (INR)	1.3 ± 0.4	1.3 ± 0.3	0.762
Antimicrobial therapy after diagnosis, N (%)			
Carbapenems	68 (73.9)	49 (60.5)	0.06
β-lactam and/or β-lactamase inhibitor	54 (58.7)	52 (64.2)	0.459
Cephalosporins	11 (12)	6 (7.4)	0.316
Fluoroquinolone	19 (20.7)	16 (19.8)	0.883
Aminoglycoside	9 (9.8)	25 (30.9)	< 0.000
Fosfomycin	7 (7.6)	17 (21.0)	0.011
Tigecycline	11 (12.0)	46 (56.8)	< 0.000
Polymyxin B	9 (9.8)	26 (32.1)	< 0.000
Ceftazidime-avibactam	5 (5.4)	32 (39.5)	< 0.000
Combination therapy	60 (65.2)	67 (82.7)	0.009
30-day mortality, N (%)	12 (13.0)	30 (37.0)	< 0.000

BSI-Kpn, bloodstream infection caused by *K pneumoniae*; CRKP, Carbapenem-resistant *K. pneumoniae*; CSKP, carbapenem-sensitive *K*. *pneumoniae*

### Antibiotic resistance and virulence profiles

A summary of 22 antibiotics MIC against BSI-Kpn is shown in Table 2. The lowest non-sensitive rates in the CRKP group were for tigecycline (4.9%) and polymyxin B (8.6%). The *bla*_KPC_ (96.3%, 78/81) (77 *bla*_KPC-2_ and 1 *bla*_KPC-3_) were the major carbapenemase genes (Supplementary Table 1). There were three isolates were negative for carbapenemase genes, of which two isolates harbored *bla*_SHV-182_ and *bla*_SHV-67_, and other one isolate co-harbored *bla*_SHV-182_, *bla*_CTX-M-15_ and *bla*_CTX-M-65_.

**Table 2:** Minimum inhibitory concentrations of 22 antibiotics against BSI-Kpn

Antibiotics	CRKP				ESBL-Kpn				S-Kpn			
	MIC range (mg/L)	MIC_90_ (mg/L)	S (N/%)	R (N/%)	MIC range (mg/L)	MIC_90_ (mg/L)	S (N/%)	R (N/%)	MIC range (mg/L)	MIC_90_ (mg/L)	S (N/%)	R (N/%)
Aztreonam	0.125– > 64	64	3 (3.7)	78 (96.3)	0.125– > 64	64	6 (20.0)	24 (80.0)	0.03–32	1	59 (95.2)	2 (3.2)
Moxalactam	0.25– > 128	128	2 (2.5)	76 (93.8)	0.06–128	8	29 (96.7)	1 (3.3)	0.125–128	2	60 (96.8)	1 (1.6)
Cefoxitin	2– > 128	128	4 (4.9)	77 (95.1)	4–128	32	18 (60.0)	6 (20.0)	2–64	32	52 (83.9)	7 (11.3)
Cefuroxime	2– > 128	128	2 (2.5)	79 (97.5)	4– > 128	128	1 (3.3)	27 (90.0)	1–128	32	45 (72.6)	10 (16.1)
Cefazolin	0.5– > 128	128	2 (2.5)	79 (97.5)	2– > 128	128	1 (3.3)	29 (96.7)	0.25–128	128	41 (66.1)	11 (17.7)
Ceftazidime	0.12– > 64	64	2 (2.5)	79 (97.5)	1– > 64	64	8 (26.7)	19 (63.3)	0.12–32	2	58 (93.5)	3 (4.8)
Ceftriaxone	0.125– > 64	64	2 (2.5)	79 (97.5)	0.25– > 64	64	3 (10.0)	27 (90.0)	0.03–32	2	56 (90.3)	3 (4.8)
Cefepime	0.06– > 64	64	4 (4.9)	77 (95.1)	0.125–64	32	6 (20.0)	20 (66.7)	0.015–16	0.5	59 (95.2)	2 (3.2)
AMC	16– > 128	128	2 (2.5)	38 (46.9)	4–64	32	5 (16.7)	7 (23.3)	1–64	16	55 (88.7)	2 (3.2)
TZP	4– > 128	128	2 (2.5)	52 (64.2)	2– > 128	128	11 (36.7)	8 (26.7)	0.25–128	32	55 (88.7)	3 (4.8)
CSL	0.25–128	128	3 (3.7)	45 (55.6)	4–128	128	12 (40.0)	10 (33.3)	0.03–32	8	61 (98.4)	1 (1.6)
Ertapenem	0.015– > 32	32	3 (3.7)	78 (96.3)	0.004–32	0.5	27 (90.0)	3 (10.0)	0.004–0.5	0.125	62 (100.0)	0 (0.0)
Imipenem	0.125– > 32	32	3 (3.7)	78 (96.3)	0.03–32	1	30 (100.0)	0 (0.0)	0.03–1	1	62 (100.0)	0 (0.0)
Meropenem	0.015– > 32	32	3 (3.7)	78 (96.3)	0.008–32	0.25	30 (100.0)	0 (0.0)	0.008–32	0.06	62 (100.0)	0 (0.0)
Gentamicin	0.5–> 128	128	12 (14.8)	60 (74.1)	0.25–128	128	16 (53.3)	12 (40.0)	0.25–128	8	55 (88.7)	4 (6.5)
Amikacin	1– > 128	128	18 (22.2)	30 (37.0)	1–128	16	28 (93.3)	2 (6.7)	0.25–8	8	62 (100.0)	0 (0.0)
Levofloxacin	0.06– > 32	32	2 (2.5)	78 (96.3)	0.25– > 32	32	4 (13.3)	16 (53.3)	0.03–32	16	41 (66.1)	4 (6.5)
Ciprofloxacin	0.015– > 32	32	2 (2.5)	79 (97.5)	1–128	32	3 (10.0)	26 (86.7)	0.004–32	4	46 (74.2)	13 (21.0)
Fosfomycin	2–256	256	55 (67.9)	19 (23.5)	0.5–64	16	30 (100.0)	0 (0.0)	0.25–256	16	59 (95.2)	1 (1.6)
SXT	0.015– > 8	8	39 (48.1)	42 (51.9)	0.015– > 8	8	5 (16.7)	25 (83.3)	0.015–8	8	54 (87.1)	8 (12.9)
Tigecycline	0.125–4	2	77 (95.1)	0 (0.0)	0.125–4	2	29 (96.7)	0 (0.0)	0.125–2	1	62 (100.0)	0 (0.0)
Polymyxin B	0.25–32	2	74 (91.4)	7 (8.6)	0.5–8	2	29 (96.7)	1 (3.3)	0.5–16	2	59 (95.2)	3 (4.8)

MIC, minimum inhibitory concentration; S, susceptible; R, resistant; CRKP, Carbapenem-resistant *K. pneumoniae*; ESBL-Kpn, extended-spectrum β-lactamases producing *K. pneumoniae*; S-Kpn, susceptible *K. pneumoniae*; AMC, amoxicillin-clavulanic acid; TZP, piperacillin-tazobactam; CSL, cefoperazone-sulbactam; SXT, Trimethoprim-sulfamethoxazol

In total, all ESBL-Kpn isolates were susceptible to imipenem, meropenem, fosfomycin. The susceptible rates of amoxicillin-clavulanic acid, piperacillin-tazobactam and cefoperazone-sulbactam for ESBL-Kpn were less than 40%. *bla*_CTX-M_ genes were detected in 22 of 62 ESBL-producing isolates: 10 (45.5%) were *bla*_CTX-M-3_, 6 (27.3%) were *bla*_CTX-M-15_, 3 (13.6%) were *bla*_CTX-M-14_, 2 were (9.1%) *bla*_CTX-M-27_, and 1 (4.5%) was *bla*_CTX-M-55_ (Supplementary Table 1).

Among 62 S-Kpn isolates, more than 90% isolate showed susceptibility to aztreonam, moxalactam, third-generation cephalosporins, carbapenems, cefoperazone-sulbactam, amikacin, fosfomycin, tigecycline and polymyxin B. A portion of S-Kpn isolates (90.3%, 56/62) harbored *bla*_SHV_ genes, of which 9 harbored *bla*_SHV-190_, 9 harbored *bla*_SHV-98_, and 6 harbored *bla*_SHV-67_ (Supplementary Table 1).

Virulence genes, such as *all*, *clb*, *clbK*, *ompA*, *wcaJ*, *ybtT*, were more common in CSKP isolates, while *rmpA2*, *sciN*, *iucA-D*, *clpV*, *icmF*, *hcp*, *fimB*, *fepC*, *ybtQ*, *qacE* were identified in more CRKP isolates (P < 0.05) (Supplementary Table 1).

### Phylogenetic analysis

Among 81 BSI-CRKP isolates, ST11 was the predominant clone, accounting for 84.0% (N = 68), by ST15 (8/81, 9.9%). However, BSI-CSKP was sporadic and ST23 was the most common clone (12/92, 13.0%). There were five O-loci (Ols, outer lipopolysaccharide synthesis loci) in CRKP group, of which O2v1 (60/81, 74.1%) was the most prevalent, followed by O2v2 (9/81, 11.1%) and OL101 (9/81, 11.1%). By contrast, O2v2 (54/92, 58.7%) was the predominant OL in CSKP group. CRKP group included 9 K-loci (KLs, capsule synthesis loci), KL64 (54/81, 66.7%) was predominant, followed by KL47 (10/81, 12.3%). The two most common KLs in CSKP group were KL2 (24/92, 26.1%) and KL1 (14/92, 15.2%).

**Fig. 1:**
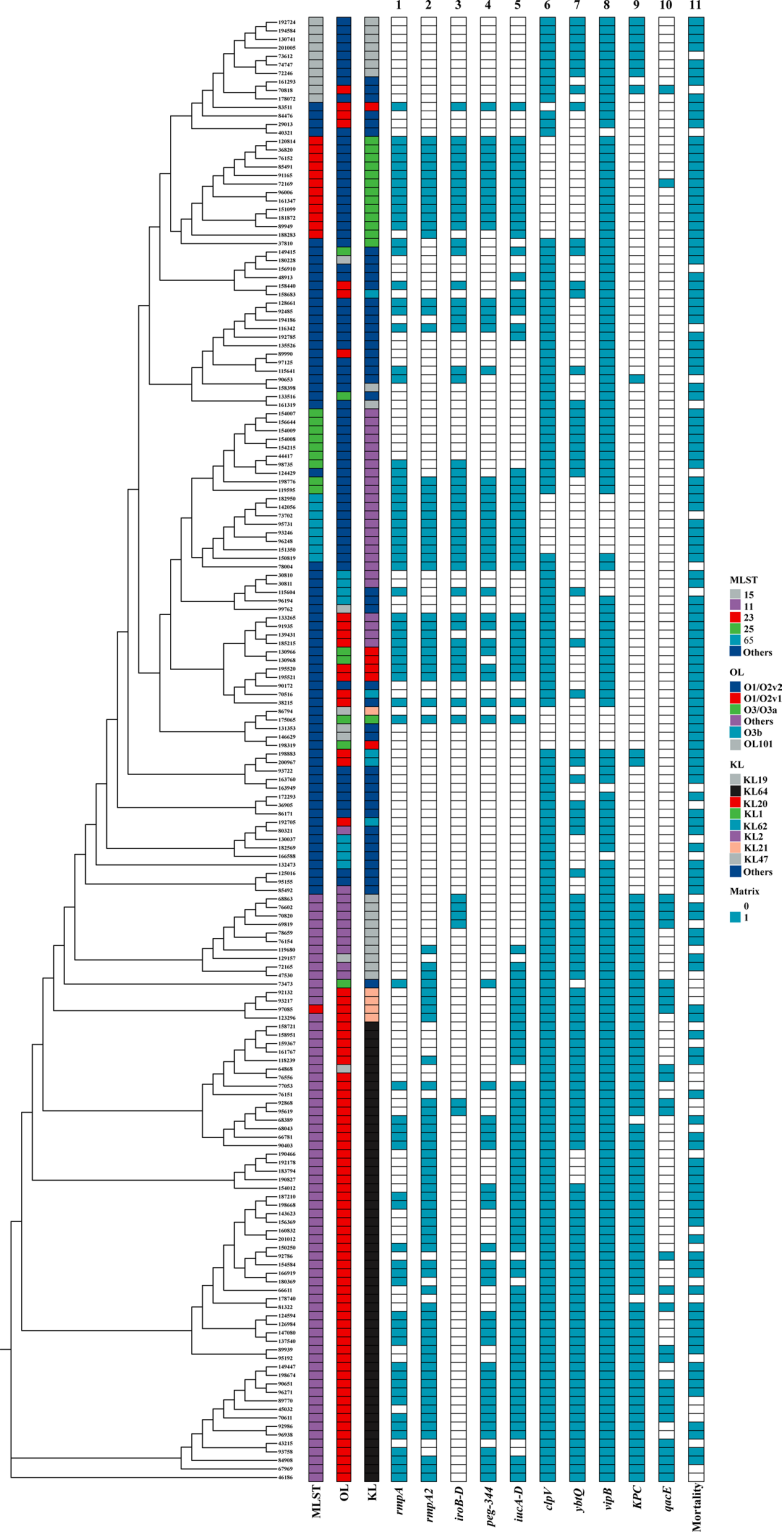
Phylogenetic analysis of 173 BSI-Kpn isolates collected in this study

Among 68 ST11-CRKP isolates, ST11-O2v1:KL64 was the most common serotypes cluster (77.9%, 53/68), followed by ST11-OL101: KL47 (13.2%, 9/68). Except for one isolate belonged to ST15-O2v1:KL19, other seven isolates were ST15-O2v2:KL19 in ST15-CRKP. All ST11-O2v1:KL64 isolates were clustered in one highly related clade of the phylogenetic tree, which is also clustered with one sublineage of ST11-OL101: KL47 and one sublineage ST11-O2v1:KL21. ST15-O2v2:KL19 in CRKP was roosted with ST15-CSKP (Fig. 1). Among CSKP, ST23-O2v2:KL1 was the predominant serotypes cluster, followed by ST25-O2v2:KL2 and ST65-O2v2:KL2, which ST25-O2v2:KL2 and ST65-O2v2:KL2 clustered together to form a branching clade and ST23-O2v2:KL1 clustered into another sublineage.

### Subpopulations of immunocyte

**Fig. 2:**
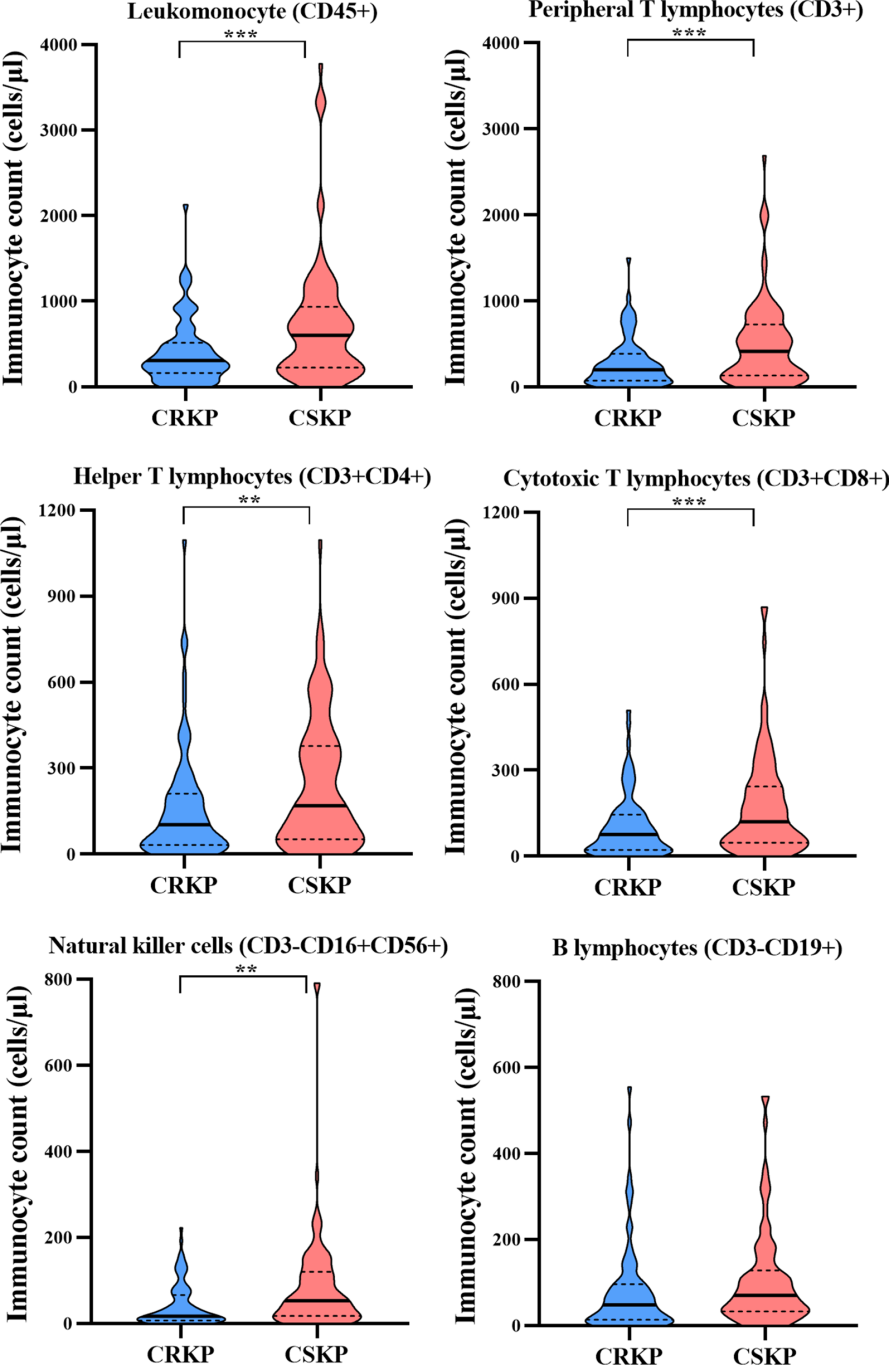
Subpopulations of immunocyte between CRKP and CSKP groups. N, Normal; D, decrease

**Fig. 3:**
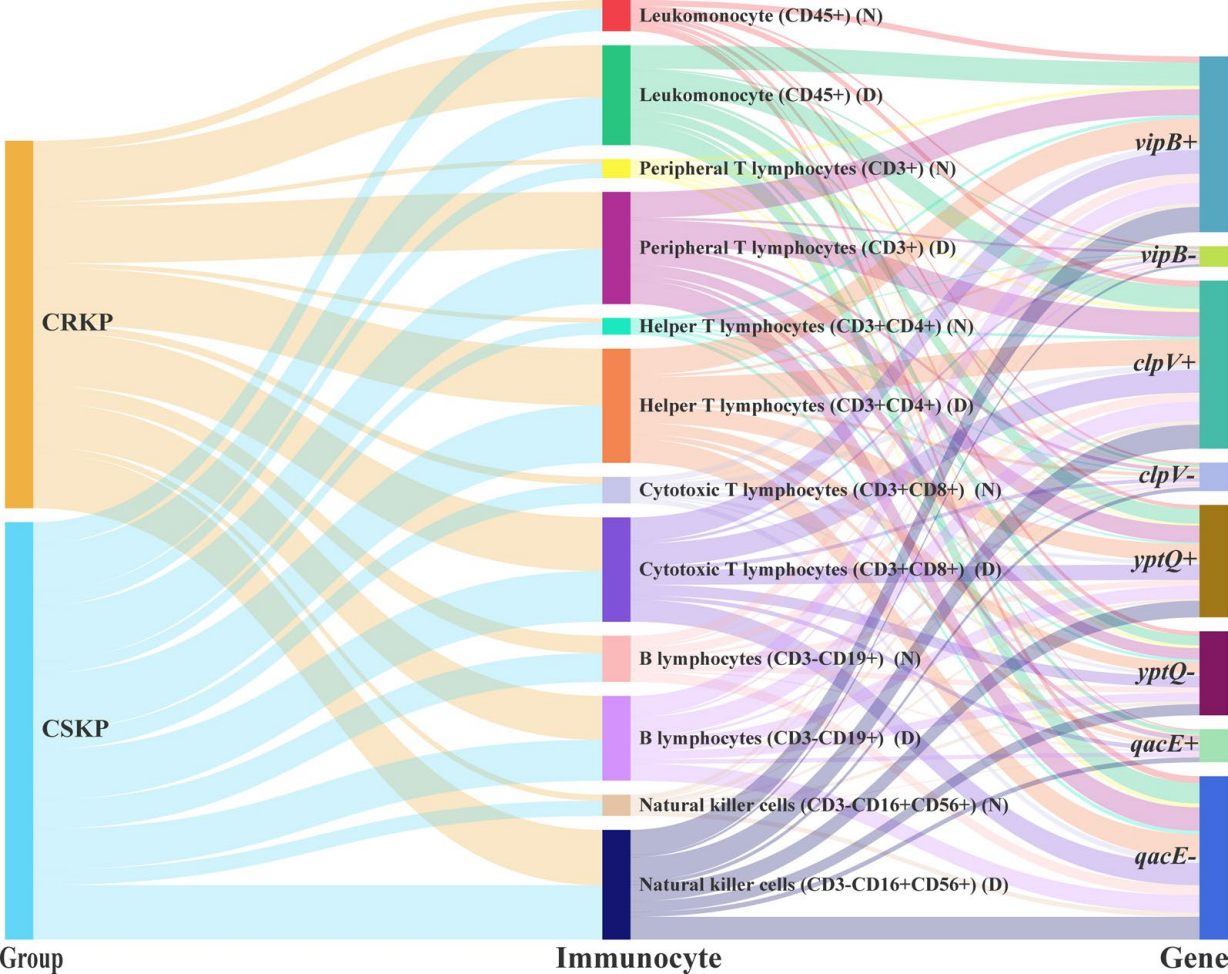
Overview of immunocyte, carbapenem resistance, and virulence genes among BSI-Kpn patients

Absolute number of leukomonocyte (CD45 +) in CRKP and CSKP were 309.0 (161.0–514.0) and 600.5 (226.5–934.3) cells/μl, respectively (Supplementary Table 2). The absolute numbers of mature peripheral T lymphocytes (CD3 +), helper T lymphocytes (CD3 + CD4 +), cytotoxic T lymphocytes (CD3 + CD8 +), and natural killer cells (CD3-CD16 + CD56 +) in patients with CRKP were significantly lower than that in CSKP (P < 0.01) (Fig. 2). The proportion of natural killer cells in the death group decreased more significantly (P = 0.041) (Supplementary Fig. 1). Among CRKP isolates, the level of cytotoxic T lymphocytes (CD3 + CD8 +) in ST11-O2v1:KL64 is higher than other serotypes and STs combinations (Supplementary Table 3). The B lymphocytes (CD3-CD19 +) showed the lowest level in ST11-O2v1:KL64. However, the difference of B lymphocytes (CD3-CD19 +) level between the death group and the survival group is not significant whether in CRKP or CSKP. The level of immunocyte in isolates co-harbored *clpV*-*ybtQ-qacE* were lower than that in isolates harbored one of *clpV*, *ybtQ* or *qacE* and without these three genes (Fig. 3 and Supplementary Table 4).

### Risk factors for 30-day mortality in enrolled patients

**Fig. 4:**
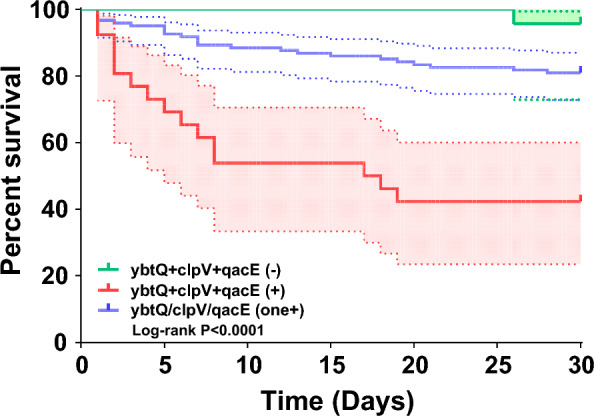
Kaplan–Meier survival estimates for patients with BSI-Kpn co-harbored *clpV*-*ybtQ*-*qacE,* harbored one of *clpV*, *ybtQ* or *qacE,* and without these three genes. A significant difference was found in the 30-day mortality between the 3 groups (P < 0.0001)

A total of 42 BSI-Kpn patients died, including 12 CSKP and 30 CRKP. The results of univariate analysis and logistic regression was shown in Table 3 and Supplementary Table 5. Compared to the survival group, BSI-Kpn patients with resistance genes (*bla*_KPC-like_, *bla*_TEM-1B_, *rmtB*, *dfrA-like*, *aadA*, *ramR*) and virulence genes (*AAA92657*, *clpV*, *ybtQ*, *ybtU*) caused higher mortality (P < 0.05). Notably, BSI-Kpn isolates co-harbored *clpV*, *ybtQ* and *qacE* resulted in higher 30-day mortality than isolates carried one of three or none of them (Fig. 4).

**Table 3:** Logistic regression model of isk factors for 30-day mortality

Parameters	Univariate analysis			Multivariable analysis			
				95% CI for EXP(B)			
	Death (N = 42)	Survivors (N = 131)	P-value	Sig	Exp(B)	Lower	Upper
ICU stay (days)	6.6 ± 16.5	1.2 ± 4.4	0.042	0.022	1.025	1.004	1.046
Hemodialysis	18 (42.9%)	26 (19.8%)	0.003	0.001	0.148	0.048	0.454
Urinary catheterization	35 (83.3%)	73 (55.7%)	0.001	0.028	0.290	0.096	0.876
Platelet (10E9/L)	36.0 (18.5–80.3)	113.0 (45.0–179.0)	< 0.000	0.001	1.012	1.005	1.019
APACHE-II score	14.5 (8.0–19.3)	10.0 (7.0–13.0)	0.001	0.009	0.892	0.818	0.972
*clpV-ybtQ-qacE* ( +)	16 (38.1%)	11 (8.4%)	< 0.000	0.000	0.098	0.032	0.299

Lower count of white blood cell, hemoglobin, platelet, and cholinesterase showed significant associations with 30-day mortality in a univariate analysis. In addition, use of glucocorticoid and immunosuppressant before BSI-Kpn are risk factors for 30-day mortality. Patients with longer ICU stay and more invasive procedures, such as mechanical ventilation, hemodialysis, urinary catheterization, arterial catheterization and stomach tube, had a higher mortality rate (P < 0.05). Tigecycline and combination therapy after diagnosis led to increased 30-day mortality. Further logistic regression established multivariate predictive model, demonstrating length of ICU stay, hemodialysis, urinary catheterization, platelet count, APACHE-II score and co-existence of *clpV*-*ybtQ*-*qacE* independently associated with a higher risk for 30-day mortality.

## Discussion

Although novel antibiotics have shown promising efficacy against multidrug-resistant bacterial infections, the mortality rate in BSI-CRKP remains high [2, 3]. Therefore, relying on antibiotics is not enough. Clearance of Kpn requires a complex, multi-faceted response of innate immune cells and ultimately resolved by adaptive immune cells [6, 21]. Therefore, the role of host immune responses and the interaction between BSI-Kpn and immunocyte in treating deadly infections deserves attention.

Kpn display a population structure characterized by genetic diversity, however, highly successful clonal genetic lineages were observed in CRKP, such as ST258 and ST11 [22, 23]. However, a previously prevalent subclone OL101:KL47 was replaced by O2v1:KL64 in the predominant clone ST11 [23]. In the present study, we also found ST11-O2v1:KL64 was the most common serotypes cluster in BSI-CRKP, which clustered with one sublineage of ST11-OL101: KL47 as well. The abundant genetic diversity between CRKP and CSKP may result in heterogeneity of interaction with host immune responses [24].

Several studies demonstrated that Kpn, especially for CRKP, could frustrate normal immune responses, capitalize upon states of immunocompromise, and cause infections with high mortality [21, 25]. Our results demonstrated the absolute numbers of tested immunocyte in patients with CRKP were significantly lower than that in CSKP (P < 0.01). Previous study found Kpn stimulated highly purified human blood B cells [26]. Our study further demonstrated the B lymphocytes (CD3-CD19 +) in ST11-O2v1:KL64 showed the lowest level. Therefore, genetic diversity of BSI-Kpn impacts the outcomes of the interactions between the Kpn and each of these host immunocyte responses. Previous studies revealed bacterial factors of hypervirulent Kpn, such as capsule polysaccharides, lipopolysaccharide, type VI secretion system (T6SS) resulted in a common evasion strategy based on phagocytosis evasion, while CRKP showed heterogeneous evasion strategies, leading to prolong intracellular survival [7, 9, 27]. However, few studies have addressed the relationship between genetic diversity of Kpn and inflammatory response. To obtain comprehensive information, it will be necessary to assess the mechanism of common and unique interaction between BSI-Kpn heterogeneity and immune cells.

Although some studies have demonstrated CRKP infections were associated with BSI mortality in several clinical series, the risk factors remain inconsistent [2, 3, 28]. CD4 + /CD8 + T lymphocytes ratio < 1 was identified as independent risk factors for ICU admission patients with CRKP infections [28]. Similarly, we found the level of CD8 + in ST11-O2v1:KL64 among patients with CRKP was higher than that in other serotypes and STs combinations. The level of immunocyte in isolates co-harbored *clpV-ybtQ-qacE* were lower than that in isolates harbored one of *clpV*, *ybtQ* or *qacE* and without these three genes. Furthermore, co-existence of *clpV-ybtQ-qacE* was independently associated with a higher risk for 30-day mortality. Previous studies reported T6SS core component (*clpV*) used by *Salmonella* to survive within host macrophages [29]. *ybtQ*, encoding a transporter for the siderophore yersiniabactin, played an important role for the ability of Kpn to maintain infection in a mammalian host [30]. *qacE,* as disinfectant resistance gene, reduced biocide susceptibility [31]. Taken together, these data show correlation among virulence, antibiotic resistance and inflammatory response, which could affect the ability to clear Kpn and increase the risk of death.

This study firstly provides an insight into interactions between different phylogenetic BSI-Kpn and immunocyte subpopulations in clinic. However, there were also several limitations in the present study. First, the immune cells were not dynamically observed during the infections. Therefore, it is unable to fully elucidate the changes in host inflammatory response throughout the entire BSI process. In addition, the number of isolates in this study remains not enough. Above all, there is a pressing need to focus on the dynamics and outcomes of the interactions between BSI-Kpn heterogeneity and host immune response.

## Conclusions

In conclusion, BSI-CRKP, especially for ST11-O2v1:KL64, was associated with 30-day mortality. In addition, subpopulations of immunocyte in patients with CRKP were lower than that in other BSI-Kpn. Furthermore, co-existence of *clpV*-*ybtQ*-*qacE* in BSI-Kpn not only showed lower immunocyte count, but it also resulted in high mortality rates. Therefore, further better understanding of BSI-Kpn-host interactions is needed to develop new therapeutics.

### Supplementary Information

Additional file 1: Supplementary Fig. 1. Subpopulations of immunocyte between survivors and death groups.

Additional file 2.

## Data Availability

All sequence data has been deposited under BioProject accession No. PRJNA778807 and PRJNA1056658.
