# Supplementary figures and images for: Genomic and immunocyte characterisation of bloodstream infection caused by Klebsiella pneumoniae
Source: Ann Clin Microbiol Antimicrob. 2024 Jun 20;23:56. doi: 10.1186/s12941-024-00721-3 (PMC11191348; doi:10.1186/s12941-024-00721-3)

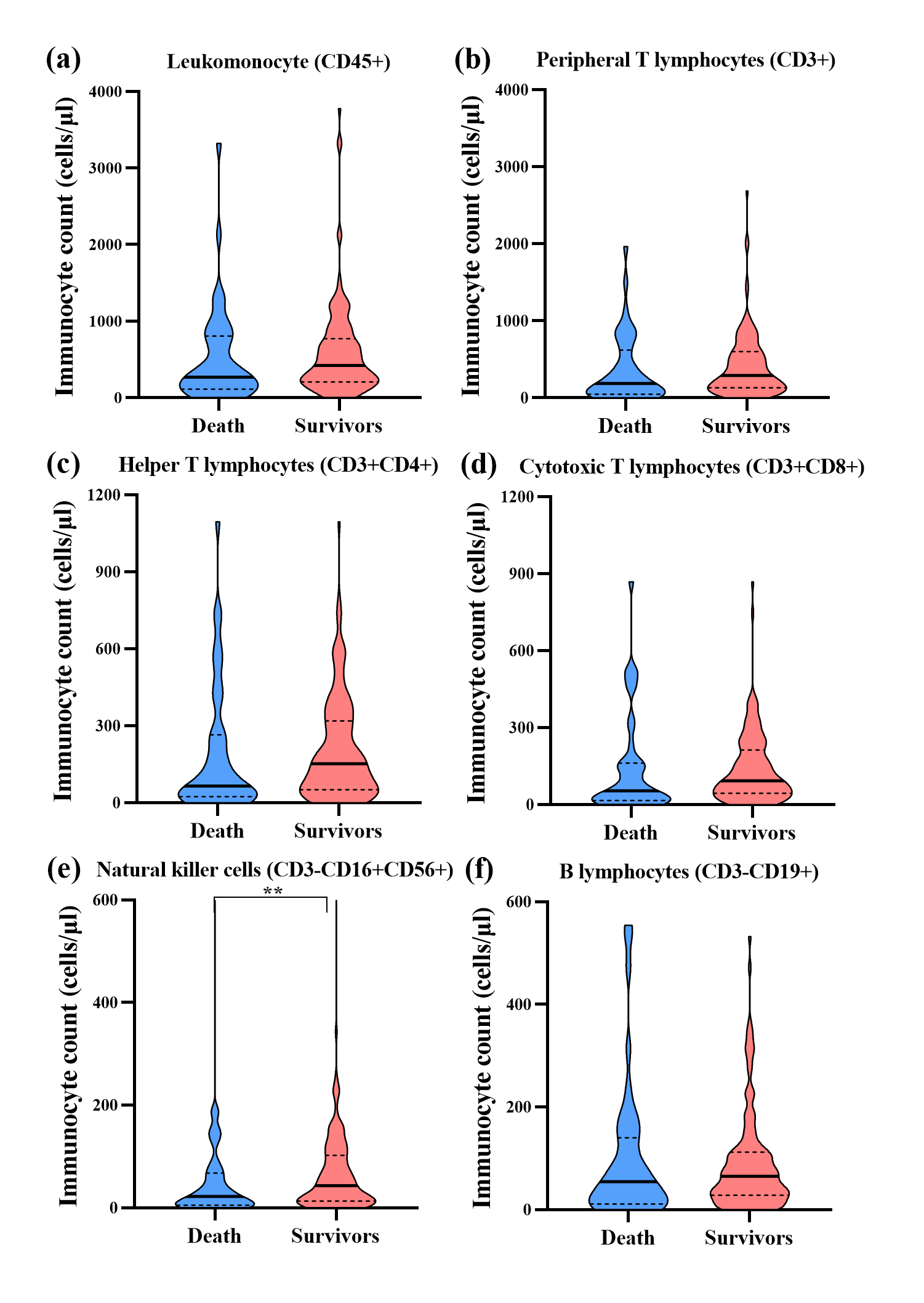

Supplement: Supplementary file 1 — Additional file 1: Supplementary Fig. 1. Subpopulations of immunocyte between survivors and death groups. [file 12941_2024_721_MOESM1_ESM.tif]
