# Supplementary material for: Genomic and immunocyte characterisation of bloodstream infection caused by Klebsiella pneumoniae
Source: Ann Clin Microbiol Antimicrob. 2024 Jun 20;23:56. doi: 10.1186/s12941-024-00721-3 (PMC11191348; doi:10.1186/s12941-024-00721-3)
Supplement: Supplementary file 2 — Additional file 2. [file 12941_2024_721_MOESM2_ESM.docx]

**Supplementary Table 1**. Resistance and virulence profiles of all BSI-Kpn.

| ***Genes*** | **CSKP (N=92)** | **CRKP (N=81)** | **P-value** |
| --- | --- | --- | --- |
| *bla_SHV-like_* | 85 (92.4%) | 68 (84.0%) | 0.083 |
| *bla_LAP-2_* | 6 (6.5%) | 35 (43.2%） | <0.000 |
| *bla_OXA-like_* | 5 (5.4%) | 2 (2.5%) | 0.323 |
| *bla_OKP-B_* | 5 (5.4%) | 0 (0%) | 0.033 |
| *bla_DHA_* | 5 (5.4%) | 5 (6.2%) | 0.836 |
| *bla_CTX-M-like_* | 22 (23.9%) | 60 (74.1%) | <0.000 |
| *bla_TEM-1B_* | 13 (14.1%) | 59 (72.8%) | <0.000 |
| *bla_KPC-like_* | 0 (0%) | 78 (96.3%) | <0.000 |
| *OqxAB* | 90 (97.8%) | 27 (33.3%) | <0.000 |
| *fos* | 92 (100%) | 81 (100%) | - |
| *qnr-like* | 25 (27.2%) | 35 (43.2%） | 0.027 |
| *rmtB* | 0 (0%) | 31 (38.3%) | <0.000 |
| *cat* | 2 (2.2%) | 26 (32.1%) | <0.000 |
| *dfrA-like* | 5 (5.4%) | 42 (51.9%) | <0.000 |
| *sul-like* | 28 (30.4%) | 44 (54.3%) | 0.001 |
| *tet(A)* | 32 (34.%) | 44 (54.3%) | 0.01 |
| *floR* | 23 (25.0%) | 2 (2.5%) | <0.000 |
| *mph(A)* | 12 (13.0%) | 12 (14.8%) | 0.737 |
| *mph(E)* | 2 (2.2%) | 5 (6.2%) | 0.183 |
| *msr(E)* | 2 (2.2%) | 6 (7.4%) | 0.102 |
| *aadA* | 20 (21.7%) | 45 (55.6%) | <0.000 |
| *aac(3)-IId* | 11 (12.0%) | 13 (16.0%) | 0.437 |
| *ant(3'')-Ia* | 4 (4.3%) | 6 (7.4%) | 0.39 |
| *aph(3')-I* | 15 (16.3%） | 7 (8.6%) | 0.131 |
| *aph(6)-Id* | 12 (13%) | 7 (8.6%) | 0.356 |
| *aac(6')-Ib* | 3 (3.3%) | 4 (4.9%) | 0.576 |
| *rmpA* | 42 (45.7%) | 30 (37.0%) | 0.251 |
| *rmpA2* | 35 (38.0%) | 51 (63.0%) | 0.001 |
| *acrAB* | 92 (100%) | 81 (100%) | - |
| *allA-D,RS* | 16 (17.4%) | 0 (0%) | <0.000 |
| *clbA-J,N,O,Q,S* | 20 (21.7%) | 0 (0%) | <0.000 |
| *clbK* | 11 (12.0%) | 3 (3.7%) | 0.047 |
| *clbL* | 18 (19.6%) | 0 (0%) | <0.000 |
| *clbP* | 19 (20.7%) | 0 (0%) | <0.000 |
| *clpV/tssH* | 67 (72.8%) | 81 (100%) | <0.000 |
| *dotU/tssL* | 61 (66.3%) | 79 (97.5%) | <0.000 |
| *hcp/tssD* | 80 (87.0%) | 81 (100%) | 0.001 |
| *icmF/tssM* | 80 (87.0%) | 81 (100%) | 0.001 |
| *impA/tssA* | 35 (38.0%) | 9 (11.1%) | <0.000 |
| *sciN/tssJ* | 71 (77.2%) | 73 (90.1%) | 0.023 |
| *tssF* | 27 (29.3%) | 2 (2.5%) | <0.000 |
| *tssG* | 30 (32.6%) | 8 (9.9%) | <0.000 |
| *vasE/tssK* | 31 (33.7%) | 2 (2.5%) | <0.000 |
| *vgrG/tssI* | 15 (16.3%) | 0 (0%) | <0.000 |
| *vipA/tssB* | 80 (87.0%) | 44 (54.3%) | <0.000 |
| *vipB/tssC* | 74 (80.4%) | 81 (100%) | <0.000 |
| *entA-C,E,F,S* | 92 (100%) | 81 (100%) | - |
| *entD* | 90 (97.8%) | 81 (100%) | 0.182 |
| *fepAB* | 92 (100%) | 81 (100%) | - |
| *fepC* | 83 (90.2%) | 81 (100%) | 0.011 |
| *fepD* | 77 (83.7%) | 81 (100%) | <0.000 |
| *fepG* | 21 (22.8%) | 0 (0%) | <0.000 |
| *fes* | 92 (100%) | 81 (100%) | - |
| *fimA,C-G,l* | 92 (100%) | 81 (100%) | - |
| *fimB* | 87 (94.6%) | 81 (100%) | 0.033 |
| *fimH* | 67 (72.8%) | 79 (97.5%) | <0.000 |
| *fimK* | 37 (40.2%) | 2 (2.5%) | <0.000 |
| *fyuA* | 5 (5.4%) | 1 (1.2%) | 0.132 |
| *fyuA/psn* | 49 (53.3%) | 78 (96.3%) | <0.000 |
| *galF* | 92 (100%) | 79 (97.5%) | 0.13 |
| *gmd* | 92 (100%) | 81 (100%) | - |
| *gndA* | 92 (100%) | 81 (100%) | - |
| *iroB-D* | 42 (45.7%) | 8 (9.9%) | <0.000 |
| *iroE* | 87 (94.6%) | 79 (97.5%) | 0.323 |
| *iroN* | 43 (46.7%) | 27 (33.3%) | 0.073 |
| *irp1* | 22 (23.9%) | 10 (12.3%) | 0.051 |
| *irp2* | 54 (58.7%) | 79 (97.5%) | <0.000 |
| *iucA-D* | 41 (44.6%) | 57 (70.4%) | 0.001 |
| *iutA* | 92 (100%) | 81 (100%) | - |
| *mrkA* | 92 (100%) | 81 (100%) | - |
| *mrkB,F,J* | 92 (100%) | 81 (100%) | - |
| *mrkC* | 27 (29.3%) | 0 (0%) | <0.000 |
| *mrkD* | 78 (84.8%) | 81 (100%) | <0.000 |
| *mrkH* | 92 (100%) | 80 (98.8%) | 0.285 |
| *mrkI* | 87 (94.6%) | 81 (100%) | 0.033 |
| *ompA* | 12 (13.0%) | 0 (0%) | 0.001 |
| *rcsA* | 90 (97.8%) | 77 (95.1%) | 0.321 |
| *rcsB* | 92 (100%) | 81 (100%) | - |
| *rfbAB* | 23 (25.0%) | 0 (0%) | <0.000 |
| *rfbK1* | 70 (76.1%) | 63 (77.8%) | 0.792 |
| *ugd* | 90 (97.8%) | 81 (100%) | 0.182 |
| *wcaJ* | 13 (14.1%) | 1 (1.2%) | 0.002 |
| *yagZ/ecpA* | 74 (80.4%) | 12 (14.8%) | <0.000 |
| *ybtA,E,S* | 54 (58.7%) | 79 (97.5%) | <0.000 |
| *ybtP* | 27 (29.3%) | 0 (0%) | <0.000 |
| *ybtQ* | 25 (27.2%) | 74 (91.4%) | <0.000 |
| *ybtT* | 26 (28.3%) | 11 (13.6%) | 0.019 |
| *ybtU* | 33 (35.9%) | 69 (85.2%) | <0.000 |
| *ybtX* | 34 (37.0%) | 5 (6.2%) | <0.000 |
| *peg-344* | 36 (39.1%) | 31 (38.3%) | 0.908 |
| *qacE* | 1 (1.1%) | 28 (34.6%) | <0.000 |
| *AAA92657* | 0 (0%) | 25 (30.9%) | <0.000 |

BSI-Kpn, bloodstream infection caused by *K pneumoniae*; CRKP, Carbapenem-resistant *K. pneumoniae*; CSKP, carbapenem-sensitive K. pneumoniae.

**Supplementary Table 2.** Subpopulations of immunocyte in BSI-Kpn patients.

| **Subpopulations of immunocyte (cells/μl)** | **CSKP (N=92)** | | | **CRKP (N=81)** | | | **P-value** |
| --- | --- | --- | --- | --- | --- | --- | --- |
|  | Death (N=12) | Survivors (N=80) | P-value | Death (N=30) | Survivors (N=51) | P-value |  |
| Leukomonocyte (CD45+) | 397.5 (187.0-886.8) | 627.5 (226.5-941.5) | 0.639 | 257.0 (100.0-743.5) | 315.0 (192.0-503.0) | 0.584 | <0.000 |
| Mature peripheral T lymphocytes (CD3+) | 262.5 (48.5-828.9) | 435.0 (143.0-727.3) | 0.554 | 132.0 (46.3-471.3) | 229.0 (103.0-364.0) | 0.425 | <0.000 |
| Helper T lymphocytes (CD3+CD4+) | 101.0 (24.0-375.0) | 171.5 (57.5-377.3) | 0.289 | 63.5 (24.0-259.8) | 110.0 (40.0-206.0) | 0.699 | 0.012 |
| Cytotoxic T lymphocytes (CD3+CD8+) | 114.0 (30.3-497.8) | 120.5 (51.3-242.0) | 0.963 | 47.5 (11.5-151.0) | 80.0 (32.0-131.0) | 0.387 | 0.001 |
| Natural killer cells (CD3-CD16+CD56+) | 28.5 (10.0-144.0) | 53.5 (19.5-113.8) | 0.582 | 16.0 (4.8-62.8) | 19.0 (10.0-78.0) | 0.246 | 0.002 |
| B lymphocytes (CD3-CD19+) | 45.5 (30.3-168.0) | 72.5 (34.3-121.5) | 0.668 | 59.0 (5.5-136.0) | 43.0 (14.0-91.0) | 0.762 | 0.11 |

BSI-Kpn, bloodstream infection caused by K pneumoniae; CRKP, Carbapenem-resistant K. pneumoniae; CSKP, carbapenem-sensitive K. pneumoniae.

**Supplementary Table 3.** Subpopulations of immunocyte in BSI-Kpn patients with different serotypes and STs combinations.

| **Category** | **Leukomonocyte (CD45+)** | **Mature peripheral T lymphocytes (CD3+)** | **Helper T lymphocytes (CD3+CD4+)** | **Cytotoxic T lymphocytes (CD3+CD8+)** | **Natural killer cells (CD3-CD16+CD56+)** | **B lymphocytes (CD3-CD19+)** |
| --- | --- | --- | --- | --- | --- | --- |
| ST11-O2v1:KL64 (N=53) | 315.0 (175.0-568.5) | 219.0 (82.5-413.0) | 99.0 (32.5-232.5) | 80.0 (23.5-154.0) | 19.0 (6.0-77.5) | 42.0 (10.5-98.0) |
| Others CRKP (N=28) | 285.5 (134.8-461.5) | 199.5 (55.0-312.0) | 110.0 (30.0-201.3) | 50.0 (14.3-109.5) | 17.0 (8.0-40.0) | 52.5 (20.3-95.5) |
| CSKP (N=92) | 600.5 (226.5-934.3) | 414.5 (134.0-727.3) | 169.5 (52.3-377.3) | 120.5 (47.5-243.5) | 53.0 (17.5-120.3) | 70.5 (33.0-128.0) |
| P-value | 0.002 | 0.002 | 0.023 | 0.004 | 0.003 | 0.053 |
| P-value (ST11-O2v1:KL64 VS Others CRKP) | 0.379 | 0.235 | 0.496 | 0.161 | 0.595 | 0.677 |
| P-value (ST11-O2v1:KL64 VS CSKP) | 0.013 | 0.023 | 0.067 | 0.035 | 0.013 | 0.026 |

BSI-Kpn, bloodstream infection caused by K pneumoniae; CRKP, Carbapenem-resistant K. pneumoniae; CSKP, carbapenem-sensitive K. pneumoniae.

**Supplementary Table 4.** Subpopulations of immunocyte in BSI-Kpn isolates co-harbored *clpV*, *ybtQ* or *qacE*.

| **Category** | **Leukomonocyte (CD45+)** | **Mature peripheral T lymphocytes (CD3+)** | **Helper T lymphocytes (CD3+CD4+)** | **Cytotoxic T lymphocytes (CD3+CD8+)** | **Natural killer cells (CD3-CD16+CD56+)** | **B lymphocytes (CD3-CD19+)** |
| --- | --- | --- | --- | --- | --- | --- |
| *clpV-ybtQ-qacE* (+) (N=27) | 252.0 (115.0-715.0) | 184.0 (49.0-501.0) | 62.0 (21.0-280.0) | 67.0 (12.0-270.0) | 27.0 (8.0-62.0) | 33.0 (6.0-85.0) |
| *clpV* or *ybtQ* or *qacE* (+) (N=23) | 766.0 (637.0-1104.0) | 567.0 (468.0-843.0) | 375.0 (278.0-535.0) | 184.0 (93.0-302.0) | 79.0 (54.0-143.0) | 101.0 (44.0-171.0) |
| *clpV-ybtQ-qacE* (-) (N=123) | 361.0 (189.0-691.0) | 233.0 (94.0-489.0) | 122.0 (39.0-237.0) | 82.0 (35.0-160.0) | 29.0 (8.0-83.0) | 62.0 (24.0-112.0) |
| P-value | 0.003 | 0.002 | 0.000 | 0.024 | 0.007 | 0.023 |

BSI-Kpn, bloodstream infection caused by K pneumoniae.

**Supplementary Table 5. Univariate analysis of risk factors for 30-day mortality**

| **Parameter/Category** | **Death (N=42)** | **Survivors (N=131)** | **P-value** |
| --- | --- | --- | --- |
| Resistance genes, N (%) | | | |
| *bla_SHV-like_* | 35 (83.3%) | 118 (90.1%) | 0.234 |
| *bla_LAP-2_* | 14 (33.3%) | 27 (20.6%) | 0.092 |
| *bla_OXA-like_* | 1 (2.4%) | 6 (4.6%) | 0.529 |
| *bla_OKP-B_* | 0 (0%) | 5 (3.8%) | 0.199 |
| *bla_DHA_* | 3 (7.1%) | 7 (5.3%) | 0.664 |
| *bla_CTX-M-like_* | 25 (59.5%) | 57 (43.5%) | 0.071 |
| *bla_TEM-1B_* | 23 (54.8%) | 49 (37.4%) | 0.047 |
| *bla_KPC-like_* | 29 (69.0%) | 49 (37.4%) | 0.001 |
| *OqxAB* | 22 (52.4%) | 95 (72.5%) | 0.015 |
| *fos* | 42 (100.0%) | 131 (100.0%) | - |
| *qnr-like* | 15 (35.7%) | 45 (34.4%) | 0.872 |
| *rmtB* | 14 (33.3%) | 17 (13.0%) | 0.003 |
| *cat* | 6 (14.3%) | 22 (16.8%) | 0.701 |
| *dfrA-like* | 19 (45.2%) | 28 (21.4%) | 0.002 |
| *sul-like* | 22 (52.4%) | 50 (38.2%) | 0.104 |
| *tet(A)* | 21 (50.0%) | 55 (42.0%) | 0.362 |
| *floR* | 7 (16.7%) | 18 (13.7%) | 0.639 |
| *mph(A)* | 8 (19.0%) | 16 (12.2%) | 0.265 |
| *mph(E)* | 3 (7.1%) | 7 (5.3%) | 0.242 |
| *msr(E)* | 4 (9.5%) | 4 (3.1%) | 0.082 |
| *aadA* | 22 (52.4%) | 43 (32.8%) | 0.023 |
| *aac(3)-IId* | 6 (14.3%) | 18 (13.7%) | 0.929 |
| *ant(3'')-Ia* | 1 (2.4%) | 9 (6.9%) | 0.278 |
| *aph(3')-I* | 7 (16.7%) | 15 (11.5%) | 0.377 |
| *aph(6)-Id* | 8 (19.0%) | 11 (8.4%) | 0.055 |
| *aac(6')-Ib* | 3 (7.1%) | 4 (3.1%) | 0.242 |
| Virulence genes, N (%) | | | |
| *rmpA* | 15 (35.7%) | 57 (43.5%) | 0.372 |
| *rmpA2* | 21 (50.0%) | 65 (49.6%) | 0.966 |
| *acrAB* | 42 (100.0%) | 131 (100.0%) | - |
| *allA-D,RS* | 0 (0%) | 16 (12.2%) | 0.017 |
| *clbA-J,N,O,Q,S* | 1 (2.4%) | 19 (14.5%) | 0.033 |
| *clbK* | 1 (2.4%) | 13 (9.9%) | 0.119 |
| *clbL* | 1 (2.4%) | 17 (13.0%) | 0.05 |
| *clbP* | 1 (2.4%) | 18 (13.7%) | 0.04 |
| *clpV/tssH* | 41 (97.6%) | 107 (81.7%) | 0.011 |
| *dotU/tssL* | 36 (85.7%) | 104 (79.4%) | 0.364 |
| *hcp/tssD* | 41 (97.6%) | 120 (91.6%) | 0.182 |
| *icmF/tssM* | 41 (97.6%) | 120 (91.6%) | 0.182 |
| *impA/tssA* | 9 (21.4%) | 35 (26.7%） | 0.493 |
| *sciN/tssJ* | 37 (88.1%) | 108 (82.4%) | 0.333 |
| *tssF* | 2 (4.8%) | 27 (20.6%) | 0.017 |
| *tssG* | 6 (14.3%) | 32 (24.4%) | 0.167 |
| *vasE/tssK* | 5 (11.9%) | 28 (21.4%) | 0.174 |
| *vgrG/tssI* | 2 (4.8%) | 13 (9.9%) | 0.301 |
| *vipA/tssB* | 27 (64.3%) | 97 (74.0%) | 0.222 |
| *vipB/tssC* | 37 (88.1%) | 118 (90.1%) | 0.714 |
| *entA-C,E,F,S* | 42 (100.0%) | 131 (100.0%) | - |
| *entD* | 42 (100.0%) | 129 (98.5%) | 0.421 |
| *fepAB* | 42 (100.0%) | 131 (100.0%) | - |
| *fepC* | 40 (95.2%) | 124 (94.7%) | 0.883 |
| *fepD* | 40 (95.2%) | 118 (90.1%) | 0.301 |
| *fepG* | 2 (4.8%) | 10 (7.6%) | 0.093 |
| *fes* | 42 (100.0%) | 131 (100.0%) | - |
| *fimA,C-G,l* | 42 (100.0%) | 131 (100.0%) | - |
| *fimB* | 42 (100.0%) | 126 (96.2%) | 0.199 |
| *fimH* | 37 (88.1%) | 109 (83.2%) | 0.447 |
| *fimK* | 5 (11.9%) | 34 (26.0%) | 0.058 |
| *fyuA* | 2 (4.8%) | 4 (3.1%) | 0.598 |
| *fyuA/psn* | 33 (78.6%) | 94 (71.8%) | 0.384 |
| *galF* | 41 (97.6%) | 130 (99.2%) | 0.393 |
| *gmd* | 1 (2.4%) | 4 (3.1%) | 0.096 |
| *gndA* | 42 (100.0%) | 131 (100.0%) | - |
| *iroB-D* | 10 (23.8%) | 40 (30.5%) | 0.403 |
| *iroE* | 42 (100.0%) | 124 (94.7%) | 0.126 |
| *iroN* | 12 (28.6%) | 58 (44.3%) | 0.071 |
| *irp1* | 4 (9.5%) | 28 (21.4%) | 0.085 |
| *irp2* | 35 (83.3%) | 98 (74.8%) | 0.254 |
| *iucA-D* | 25 (59.5%) | 73 (55.7%) | 0.666 |
| *iutA* | 42 (100.0%) | 131 (100.0%) | - |
| *mrkA* | 42 (100.0%) | 131 (100.0%) | - |
| *mrkB,F,J* | 42 (100.0%) | 131 (100.0%) | - |
| *mrkC* | 1 (2.4%) | 26 (19.8%) | 0.007 |
| *mrkD* | 41 (97.6%) | 118 (90.1%) | 0.119 |
| *mrkH* | 42 (100.0%) | 130 (99.2%) | 0.57 |
| *mrkI* | 42 (100.0%) | 126 (96.2%) | 0.199 |
| *ompA* | 1 (2.4%) | 11 (8.4%) | 0.182 |
| *rcsA* | 39 (92.9%) | 128 (97.7%) | 0.135 |
| *rcsB* | 42 (100.0%) | 131 (100.0%) | - |
| *rfbAB* | 1 (2.4%) | 22 (16.8%) | 0.017 |
| *rfbK1* | 32 (76.2%) | 101 (77.1%） | 0.903 |
| *ugd* | 42 (100.0%) | 129 (98.5%) | 0.421 |
| *wcaJ* | 1 (2.4%) | 13 (9.9%) | 0.119 |
| *yagZ/ecpA* | 13 (31.0%) | 73 (55.7%) | 0.005 |
| *ybtA,E,S* | 35 (83.3%) | 98 (74.8%) | 0.254 |
| *ybtP* | 2 (4.8%) | 25 (19.1%) | 0.026 |
| *ybtQ* | 31 (73.8%) | 68 (51.9%) | 0.013 |
| *ybtT* | 6 (14.3%) | 31 (23.7%) | 0.197 |
| *ybtU* | 31 (73.8%) | 71 (54.2%) | 0.025 |
| *ybtX* | 5 (11.9%) | 34 (26.0%) | 0.058 |
| *peg-344* | 14 (33.3%) | 53 (40.5%) | 0.409 |
| *AAA92657* | 10 (23.8%) | 15 (11.5%) | 0.047 |
| Gender, male, N (%) | 27 (64.3%) | 86 (65.6%) | 0.872 |
| Age (years) | 57.0 (39.0-65.3) | 53.0 (44.0-62.0) | 0.803 |
| APACHE-II score | 14.5 (8.0-19.3） | 10.0 (7.0-13.0) | 0.001 |
| SOFA score | 8.8±3.7 | 5.4±4.0 | <0.000 |
| CRKP, N (%) | 30 (71.4%) | 51 (38.9%) | <0.000 |
| Prior chemotherapy or radiotherapy, N (%) | 9 (21.4%) | 24 (18.3%) | 0.656 |
| Prior corticosteroid therapy, N (%) | 23 (54.8%) | 43 (32.8%) | 0.011 |
| Prior immunosuppressant use, N (%) | 17 (40.5%) | 32 (24.4%) | 0.045 |
| Comorbid illness, N (%) | | | |
| Diabetes | 6 (14.3%) | 27 (20.6%) | 0.364 |
| Hepatitis | 18 (42.9%) | 36 (27.5%) | 0.061 |
| Tumor | 13 (31.0%) | 32 (24.4%) | 0.402 |
| Hypertension | 12 (28.6%) | 48 (36.6%) | 0.339 |
| Coronary heart disease | 2 (4.8%) | 3 (2.3%) | 0.405 |
| Cerebral infarction | 0 (0%) | 8 (6.1%) | 0.101 |
| Renal insufficiency | 10 (23.8%) | 28 (21.4%) | 0.74 |
| Trauma | 3 (7.1%) | 8 (6.1%) | 0.811 |
| Organ transplant | 15 (35.7%) | 35 (26.7%） | 0.263 |
| ICU stay (days) | 6.6±16.5 | 1.2±4.4 | 0.042 |
| Invasive procedures, N (%) | | | |
| Mechanical ventilation | 28 (66.7%) | 62 (47.3%) | 0.029 |
| Hemodialysis | 18 (42.9%) | 26 (19.8%) | 0.003 |
| Urinary catheterization | 35 (83.3%) | 73 (55.7%) | 0.001 |
| Arterial catheterization | 37 (88.1%) | 77 (58.8%) | <0.000 |
| Stomach tube | 29 (69.0%) | 60 (45.8%) | 0.009 |
| Bronchofibroscope use | 9 (21.4%) | 15 (11.5%) | 0.104 |
| Wound drainage tube use | 35 (83.3%) | 101 (77.1%） | 0.256 |
| Laboratory examination | | | |
| White blood cell (10E9/L) | 7.2±7.2 | 9.7±6.5 | 0.035 |
| Neutrophil percentage (%) | 90.8 (79.2-95.3) | 90.4 (82.7-94.1) | 0.236 |
| Hemoglobin (g/L) | 82.6±26.4 | 94.0±27.0 | 0.018 |
| Platelet (10E9/L) | 36.0 (18.5-80.3) | 113.0 (45.0-179.0) | <0.000 |
| Hypersensitivity C reactive protein (mg/L) | 93.2±89.6 | 89.8±68.2 | 0.794 |
| Procalcitonin (ng/ml) | 3.8 (1.0-11.6) | 2.4 (0.7-13.1) | 0.737 |
| Albumin (g/L) | 32.7 (28.425-37.2) | 34.4 (31.2-38.4) | 0.244 |
| Alanine transaminase (U/L) | 54.5 (15.0-175.0) | 38.0 (17.0-118.0) | 0.149 |
| Aspartate aminotransferase (U/L) | 56.5 (19.5-131.75) | 38.0 (19.0-83.0) | 0.272 |
| Cholinesterase (U/L) | 2982.5±1599.9 | 3928.5±1780.9 | 0.003 |
| Total bilirubin (μmol/L) | 39.0 (13.6-171.2) | 14.2 (8.0-48.8) | 0.657 |
| Serum creatinine (μmol/L) | 72.5 (55.0-118.8) | 77.0 (54.0-149.0) | 0.025 |
| International normalised ratio (INR) | 1.3 (1.1-1.8) | 1.1 (1.0-1.2) | 0.002 |
| Subpopulations of immunocyte (cells/μl) | | | |
| Leukomonocyte (CD45+) | 267.0 (112.5-804.3) | 422.0 (206.0-772.0) | 0.148 |
| Mature peripheral T lymphocytes (CD3+) | 188.0 (46.8-620.25) | 291.0 (131.0-602.0) | 0.085 |
| Helper T lymphocytes (CD3+CD4+) | 65.0 (24.0-265.8) | 152.0 (51.0-319.0) | 0.117 |
| Cytotoxic T lymphocytes (CD3+CD8+) | 54.0 (16.8-162.0) | 93.0 (44.0-213.0) | 0.14 |
| Natural killer cells (CD3-CD16+CD56+) | 22.0 (5.0-67.5) | 43.0 (13.0-102.0) | 0.041 |
| B lymphocytes (CD3-CD19+) | 54.5 (11.3-140.0) | 65.0 (28.0-112.0) | 0.544 |
| Antimicrobial therapy after diagnosis, N (%) | | | |
| Carbapenems | 29 (69.0%) | 88 (67.2%) | 0.821 |
| β-lactam and/or β-lactamase inhibitor | 21 (50.0%) | 85 (64.9%) | 0.085 |
| Cephalosporins | 1 (2.4%) | 16 (12.2%) | 0.062 |
| Fluoroquinolone | 7 (16.7%) | 28 (21.4%) | 0.509 |
| Aminoglycoside | 12 (28.6%) | 22 (16.8%) | 0.095 |
| Fosfomycin | 9 (21.4%) | 15 (11.5%) | 0.104 |
| Tigecycline | 22 (52.4%) | 35 (26.7%） | 0.002 |
| Polymyxin B | 8 (19.0%) | 27 (20.6%) | 0.826 |
| Ceftazidime-avibactam | 10 (23.8%) | 27 (20.6%) | 0.66 |
| Combination therapy | 37 (88.1%) | 90 (68.7%) | 0.013 |

CRKP, Carbapenem-resistant K. pneumoniae.
